# Evaluation of immunohistochemical and gene expression of Janus kinase1 and Janus kinase3 in the skin of different clinical types of mycosis fungoides patients – Part II: reverse transcriptase–polymerase chain reaction^؆^

**DOI:** 10.1016/j.abd.2026.501300

**Published:** 2026-03-19

**Authors:** Heba Saed El-Amawy, Basma Mourad Mohamed Ali, Mohamed Labib Salem, Lamia Elgarhy

**Affiliations:** a Department of Dermatology and Venereology, Faculty of Medicine, Tanta University, Tanta, Egypt; b Department of Zoology, Faculty of Science, Tanta University, Tanta, Egypt; c Center of Excellence in Cancer Research (CECR), Tanta University, Tanta, Egypt

**Keywords:** Janus kinases, Janus kinase 1, Janus kinase 3, Mycosis fungoides, Polymerase chain reaction

## Abstract

**Background:**

Mycosis Fungoides (MF) is the commonest type of primary cutaneous T-Cell lymphomas representing about 50% of all lymphomas arising primarily in the skin. Janus kinases are non-receptor intracellular tyrosine kinases that play an important role in the pathogenesis of variant skin disorders and several hematological malignancies.

**Objective:**

The aim of this study was to investigate the gene expression of Janus Kinase-1 (JAK1) and Janus Kinase-3 (JAK3) in the skin of different types of MF patients using Real-time Quantitative Reverse Transcription Polymerase Chain Reaction (RT-PCR).

**Methods:**

The current study included 53 patients with MF, and 53 control samples. RT-PCR for JAK1 and JAK3 was done in the skin specimens obtained from patients and controls.

**Results:**

Both JAK1 and JAK3 fold changes showed stepwise upregulation in lesional MF skin than normal control skin, with statistically significant increase in MF patients than healthy controls (p < 0.001 and < 0.001 respectively). JAK1 was significantly upregulated in early-stage MF than JAK3 (p < 0.001).

**Study limitations:**

Limitations of this study include the small sample size of some mycosis fungoides variants, such as erythrodermic MF. Further studies are needed to clarify the functional role of Janus kinase signaling pathways in MF pathogenesis.

**Conclusions:**

Both JAK1 and JAK3 play a role in the pathogenesis of MF. JAK1 may have a pathogenic role, particularly in the early stage of cutaneous T-cell lymphoma. This potentiates the idea of using JAK1 and JAK3 inhibitors for MF treatment.

## Introduction

Mycosis fungoides is the most common type of primary cutaneous T-Cell Lymphoma (CTCL) accounts for 4% of all non-Hodgkin lymphoma cases. It has an unclear cause, but various hypotheses have been proposed, including genetic abnormalities, environmental and occupational exposure to certain chemicals, possible infectious etiology, and different upregulated cytokines.^1^

The clinical presentation of MF is variable, depending on the stage of the disease, either patch, plaque, or tumor stage. It is easy to confuse MF for common cutaneous disorders such as dermatitis, psoriasis, parapsoriasis, or drug eruptions. The predominant histopathological features include lymphocyte infiltration, epidermotropism with absent spongiosis, and lymphoid atypia.^2^ Although the definitive diagnosis of mycosis fungoides relies on histopathology, dermoscopy can serve as a valuable tool, not only aiding in early detection but also helping to distinguish the dermoscopic patterns across different disease stages and various clinical subtypes of MF.^3^

Janus kinase proteins are a family of intracellular non-receptor tyrosine kinases that includes JAK1, JAK2, JAK3, and Tyk2. They are involved in the transmission of variable cytokine signals through the JAK–STAT (signal transducers and activators of transcription) pathway.^4^

JAK activation and signaling have been proven to have a key role in the pathogenesis of several neoplasms and inflammatory disorders. JAK inhibitors have proven efficacy when used in the treatment of some dermatological diseases, including psoriasis and atopic dermatitis.^5^ The current work is a trial to confirm their role in the pathogenesis of this disease.

## Patients and methods

The present case-control study (approval nº 33254/07/19) included (53) MF patients after giving a written consent, diagnosed clinically and confirmed histopathologically as MF, collected from the local outpatient clinic of Dermatology and Venereology and Clinical Oncology department, Tanta university hospitals, in addition to (53) healthy subjects from healthy volunteers served as controls, matching the age and sex of MF patients. The included MF patients were newly or previously diagnosed cases of MF who did not receive any treatment for at least 3-months before the study. Pregnant or lactating females, patients with associated dermatological or systemic diseases were excluded.

All patients were subjected to complete history taking, general examination to detect any associated systemic comorbidities, routine laboratory investigations, history of MF illness, including onset, course and duration of cutaneous lesions, site of lesions and associated itching or pain.

Clinical and histopathological assessment of the studied MF patients was discussed in part 1 of the study, which evaluated JAK1 and JAK3 immunohistochemical expression.

### Real-Time quantitative reverse transcription Polymerase Chain Reaction (RT-PCR) for JAK1 and JAK3

Total RNA extraction from each Paraffin-embedded tissue was done through cutting seven 40 µm thickness tissue sections from each paraffin block and then deparaffinization in xylene to purify the skin samples using the RNeasy® FFPE Kit (Cat. nº 73504, Qiagen, Hilden, Germany) as instructed by the manufacturer. After quantification, cDNA was synthesized from 1 μg RNA using the SensiFAST™ cDNA Synthesis Kit (Meridian Bioscience, Cat. nº BIO-65053, USA). Real-time PCR was performed using the SensiFAST™ SYBR Lo-ROX Kit (Meridian Bioscience, Cat. nº BIO-94005, 500 × 20 µL Reactions, USA). U6 small nuclear RNA was used as an internal control to normalize the expression of genes. The sequences of the JAK1 and JAK3 primer sets are shown in Table 1. The procedure of real-time PCR: Initialization through a temperature of 95 °C for 2 minutes for polymerase activation; denaturation through a temperature of 95 °C for 5 seconds; annealing/extension through a temperature of 60 °C for 34 seconds; 40 cycles.

**Table 1:** The Primary sequence for the analyzed gene.

**Primer**	**Sequence (**5’-3’**)**
JAK1 F	ACTAAGAAAGCCCAGGAGTG
JAK1 R	AGGGTCCCAGAATAGATGTG
JAK3 F	CCACTCCCTCTTTGCTCTGG
JAK3 R	AAATCCTTGCGTAGCCCGAA
GAPDH F	GGATTTGGTCGTATTGGG
GAPDH R	GGAAGATGGTGATGGGATT

GAPDH, Glyceraldehyde 3-Phosphate Dehydrogenase.

### Fold change evaluation

The formula RQ = 2^−ΔΔCT^ was used to calculate the relative gene expression after normalization to glyceraldehyde 3-phosphate dehydrogenase endogenous control. Each sample had at least 3 triplicates. The mean of 3 independent triplicates was used to express the values of JAK1 and JAK3 fold change.^6^

### Statistical analysis

Data was analyzed using IBM SPSS version 20.0. (Armonk, NY: IBM Corp). Categorical data were compared using Chi-square, Fisher's Exact, or Monte Carlo tests as needed. Quantitative data comparisons employed Student *t*-test and ANOVA for normal distributions, while Mann-Whitney and Kruskal-Wallis tests (with Dunn's Post Hoc) were used for non-normal distributions. Correlations used Spearman for non-normal and Pearson for normal distributions. The Significance of the obtained results was judged at the 5% level.

## Results

The present study included 53 MF patients, aged from 5- to 70-years, with 18 patients being males (34%), and 35 being females (66%). The duration of lesions ranged from 0.08 to 25-years with a mean of 4.82 ± 5.23 years. MF patients included 22 patients (41.6%) suffering from classic MF: 10 patients with patch stage (18.9%), 9 with plaque stage (17%) and 3 with tumor stage (5.7%). The remaining 31 patients (58.5%) were: 16 patients with hypopigmented MF (30.2%), 7 with hyperpigmented MF (13.2%), 5 with poikilodermatous MF (9.4%) and 3 with erythrodermic MF (5.7%). 46 MF patients were early-stage MF (86.8%), and 7 patients had late-stage MF (13.2%). The demographic, clinical and histopathological data, with the relation between MF clinical type and the histopathological data and staging of the patients, are mentioned in detail in Supplementary Tables 1, 2 and 3.

### Results of RT-PCR of JAK1 and JAK3

**Fig. 1:**
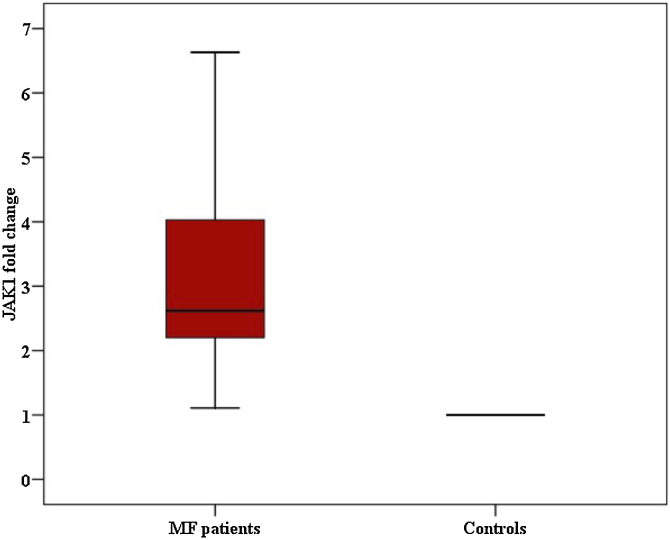
Comparison between Mycosis fungoides patients and controls according to fold change for JAK1.

**Fig. 2:**
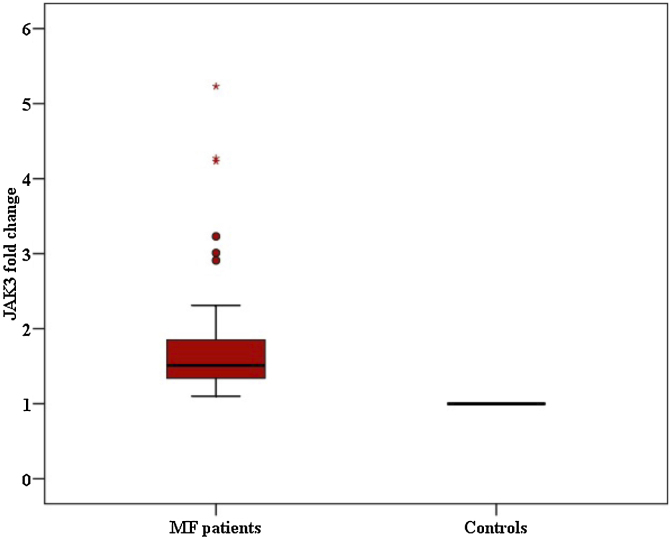
Comparison between Mycosis fungoides patients and controls according to fold change for JAK3.

Both JAK1 and JAK3 gene expression showed a stepwise upregulation in lesional MF skin than the normal control skin as follows: There was a statistically significant increase of JAK1 fold change in MF patients than healthy controls with p-value < 0.001, and there was statistically significant increase of JAK3 fold change in MF patients than healthy controls with p-value < 0.001 (Fig. 1, Fig. 2).

### JAK gene expression in different MF clinical types using RT-PCR

JAK1 gene expression in different MF clinical types

There was a statistically significant increase in JAK1 gene expression fold change in the plaque stage than the patch stage (P1 = 0.041). When comparing patch, plaque, and tumor stage MF, there was a significantly higher gene expression of JAK1 fold change in the tumor stage than patch and plaque stage MF (P7 = 0.020). JAK1 gene expression fold change was greater in tumor stage MF than hypopigmented MF, with no statistically significant difference (P4 = 0.220). There was a statistically significant increase in JAK1 gene expression fold change in hypopigmented MF when compared with hyperpigmented, poikilodermatous, and erythrodermic MF (P6 = 0.016). There were statistically significant differences regarding JAK1 gene expression fold change between the different clinical types of MF (p = 0.002), (Table 2, Supplementary Fig. 1).

**Table 2:** Relation & comparison between JAK1 and JAK3 fold change in different Mycosis Fungoides clinical types (n = 53).

Clinical type	N	JAK1 fold change		JAK3 fold change		P8
		Mean ± SD	Median (Min. – Max.)	Mean ± SD	Median (Min. – Max.)	
Classic MF (Patch stage)	**10**	2.65 ± 0.92	2.89^b^, ^c^ (1.11 – 4.03)	1.50 ± 0.35	1.40^c^, ^e^ (1.10 – 2.10)	0.028^a^
Classic MF (Plaque stage)	**9**	4.21 ± 1.69	4.29 (1.30 – 6.41)	1.61 ± 0.54	1.40^c^, ^e^ (1.14 – 2.91)	0.008^a^
Classic MF (Tumor stage)	**3**	4.41 ± 0.81	4.41(3.6 – 5.21)	4.58 ± 0.57	4.27 (4.23 – 5.23)	0.593
Hypopigmented MF	**16**	3.39 ± 1.32	2.90^c^ (2.0 – 6.63)	1.40 ± 0.19	1.40^c^, ^e^, ^f^ (1.18 – 1.91)	<0.001^a^
Hyperpigmented MF	**7**	2.45 ± 0.57	2.30^b^, ^c^ (1.91 – 3.58)	1.66 ± 0.04	1.66 (1.60 – 1.71)	0.008^a^
Poikilodermatous MF	**5**	1.79 ± 0.08	1.80^b^, ^c^, ^d^ (1.71 – 1.91)	1.92 ± 0.24	1.91 (1.65 – 2.31)	0.285
Erythrodermic MF	**3**	2.04 ± 0.67	2.20^b^, ^c^ (1.30 – 2.62)	2.50 ± 1.09	3.01 (1.25 – 3.23)	0.593
**H(p)**		20.256^a^ (0.002^a^)		20.279^a^ (0.002^a^)		
**P1**		0.041^a^		0.871		
**P2**		0.051		0.003^a^		
**P3**		0.604		0.006^a^		
**P4**		0.220		0.001^a^		
**P5**		0.118		0.038^a^		
**P6**		0.016^a^		0.001^a^		
**P7**		0.020^a^		0.023^a^		

H, H for Kruskal Wallis test, Pairwise comparison bet. each 2 groups was done using Post Hoc Test (Dunn's for multiple comparisons test); p, p-value for comparing between different categories.

P1: p-value for comparing between patch stage and plaque stage MF.

P2: p-value for comparing between patch stage and tumor stage MF.

P3: p-value for comparing between plaque stage and tumor stage MF.

P4: p-value for comparing between tumor stage and hypopigmented MF.

P5: p-value for comparing between hypopigmented MF and hyperpigmented MF.

P6: p-value for comparing between hypopigmented, hyperpigmented, *Poikilodermatous* and erythrodermic MF.

P7: p-value for comparing between Patch stage, plaque stage and tumor stage.

P8: p-value for Wilcoxon signed ranks test for comparing between JAK1 fold change and JAK3 fold change.

a Statistically significant at p ≤ 0.05.

b Significant with Classic MF (Plaque stage).

c Significant with Classic MF (Tumor stage).

d Significant with Hypopigmented MF.

e Significant with Poikilodermatous MF.

f Significant with Hyperpigmented MF.

JAK3 gene expression in different MF clinical types

There was a statistically significant increase in JAK3 gene expression fold change in tumor stage than patch stage (P2 = 0.003) and a statistically significant increase in tumor stage than plaque stage MF (P3 = 0.006). When comparing patch, plaque and tumor stage MF, there was a significantly higher JAK3 gene expression fold change in tumor stage than in the patch and plaque stage MF (P7 = 0.023). JAK3 gene expression fold change was greater in tumor stage MF than in hypopigmented MF with a statistically significant difference (P4 = 0.001). JAK3 gene expression fold change was greater in hyperpigmented MF than hypopigmented MF with a statistically significant difference (P5 = 0.038). There was a statistically significant increase in JAK3 gene expression fold change in erythrodermic MF when compared with hypopigmented, hyperpigmented, and poikilodermatous MF (P6 = 0.001). There were statistically significant differences regarding JAK3 fold change in different clinical types of MF (p = 0.002), (Table 2 and Supplementary Fig. 2).

### Comparison between JAK1 and JAK3 gene expression in different MF clinical types

In the patch and plaque stage MF, JAK1 gene expression fold change showed statistically significant increase over JAK3 fold change (p = 0.028, 0.008, respectively). In hypopigmented and hyperpigmented, JAK1 gene expression fold change showed statistically significant increase than JAK3 fold change (p < 0.001, p = 0.008, respectively) (Table 2).

### Relation between JAK1 gene expression using RT-PCR and histopathological data in MF patients

JAK1 gene expression fold change in patients with epidermotropism ranged from 1.11 to 6.63, with a mean of 3.06 ± 1.36, while in patients with absent epidermotropism, JAK1 gene expression fold change ranged from 3.60 to 4.41, with a mean of 4.01 ± 0.57, with no statistically significant difference (p = 0.264). In patients with Pautrier microabcesses in histopathology, JAK1 gene expression fold change level ranged from 1.30 to 6.41 with a mean of 4.34 ± 1.68, in comparison to the patients with absent Pautrier microabcess where the gene expression of JAK1 ranged from 1.11 to 6.63 with a mean of 2.84 ± 1.14, and there was a statistically significant increase in JAK1 gene expression fold change in patients with Pautrier microabcesses than in patients with absent Pautrier microabcesses (p = 0.006, Table 3).

**Table 3:** Relation between JAK1 & JAK3 fold change and histopathological data in Mycosis Fungoides patients (n = 53).

Histopathological data	N	JAK1 fold change		Test of sig.	p	JAK3 fold change		Test of sig.	p
		Mean ± SD	Median (Min. – Max.)			Mean ± SD	Median (Min. – Max.)		
**Epidermotropism**									
Absent	**2**	4.01 ± 0.57	4.01 (3.60 – 4.41)	U **=** 25.50	0.264	4.25 ± 0.03	4.25 (4.23 – 4.27)	U = 2.0^a^	0.006^a^
Present	**51**	3.06 ± 1.36	2.60 (1.11 – 6.63)			1.68 ± 0.68	1.51 (1.10 – 5.23		
**Dermal lymphocytes infiltrate**									
Papillary only	**50**	3.02 ± 1.34	2.60 (1.11 – 6.63)	U **=** 26.50	0.061	1.61 ± 0.46	1.51 (1.10 – 3.23)	U = 0.0^a^	<0.001^a^
Papillary and reticular	**3**	4.41 ± 0.81	4.41 (3.60 – 5.21)			4.58 ± 0.57	4.27 (4.23 – 5.23)		
**Degree of lymphocytic atypia**									
Mild (1)	**31**	3.06 ± 1.35	2.6 (1.11 – 6.63)	H = 3.024	0.220	1.54 ± 0.27	1.51 (1.11 – 2.10)	H = 2.494	0.287
Moderate (2)	**17**	2.9 ± 1.38	2.6 (1.3 – 6.41)			1.79 ± 0.67	1.60 (1.10 – 3.23)		
Severe (3)	**5**	3.98 ± 1.15	4.41(2.2 – 5.21)			3.28 ± 1.83	4.23(1.25 – 5.23)		
**Pautrier microabcess**									
Absent	**44**	2.84 ± 1.14	2.40 (1.11 – 6.63)	U **=** 85.0^a^	0.006^a^	1.7 ± 0.67	1.52 (1.1 – 4.27)	U = 179.50	0.666
Present	**9**	4.34 ± 1.68	4.50 (1.30 – 6.41)			2.19 ± 1.34	1.48 (1.14 – 5.23)		
**Epidermal atrophy**									
Absent	**48**	3.23 ± 1.35	2.89 (1.11 – 6.63)	U **=** 26.0^a^	0.002^a^	1.77 ± 0.87	1.51 (1.10 – 5.23)	U = 51.0^a^	0.034^a^
Present	**5**	1.79 ± 0.08	1.80 (1.71 – 1.91)			1.92 ± 0.24	1.91 (1.65 – 2.31)		
**Prominent vascularity**									
Absent	**44**	3.36 ± 1.33	3.20 (1.11 – 6.63)	U **=** 42.0^a^	<0.001^a^	1.73 ± 0.85	1.51 (1.10 – 5.23)	U = 120.50	0.066
Present	**9**	1.82 ± 0.41	1.80 (1.30 – 2.62)			2.03 ± 0.71	1.91 (1.20 – 3.23)		
**Basal hyperpigmentation & dermal melanophages**									
Absent	**41**	3.36 ± 1.40	3.20 (1.11 – 6.63)	U **=** 105.50^a^	0.003^a^	1.79 ± 0.94	1.40 (1.10 – 5.23)	U = 135.0^a^	0.018^a^
Present	**12**	2.17 ± 0.55	1.91 (1.71 – 3.58)			1.77 ± 0.20	1.69 (1.60 – 2.31)		

U, Mann Whitney test; H, H for Kruskal Wallis; p, p-value for comparing between different categories; N, Number of patients.

a Statistically significant at p ≤ 0.05.

Patients with the presence of epidermal atrophy in their skin lesions had a mean of JAK1 gene expression 1.79 ± 0.08, while patients with absent epidermal atrophy had a mean of JAK1 gene expression fold change 3.23 ± 1.35, with a statistically significant increase of JAK1 gene expression fold change in patients with absent epidermal atrophy (p = 0.002). Patients with the presence of prominent vascularity and hyperpigmentation in histopathology had a mean JAK1 gene expression fold change of 1.82 ± 0.41 and 2.17 ± 0.55, respectively. There was a statistically significant increase in JAK1 gene expression fold change in patients with absent prominent vascularity and absent hyperpigmentation (p < 0.001, p = 0.003, respectively), (Table 3).

### Relation between JAK3 gene expression using RT-PCR and histopathological data in MF patients

JAK3 gene expression fold change in patients with epidermotropism ranged from 1.10 to 5.23, with a mean of 1.68 ± 0.68, while patients with absent epidermotropism had a mean of 4.25 ± 0.03 with a statistically significant increase of JAK3 gene expression fold change in patients with absent epidermotropism (p = 0.006). JAK3 gene expression fold change level in patients with papillary and reticular dermal lymphocytes ranged from 4.23 to 5.23 with a mean of 4.58 ± 0.57, compared to patients with papillary dermal lymphocytes only, which had a mean of 1.61 ± 0.46, with a statistically significant increase in patients with papillary and reticular dermal infiltrate (p < 0.001, Table 3).

Patients with the presence of epidermal atrophy in their skin lesions had a mean of JAK3 gene expression 1.92 ± 0.24, while patients with absent epidermal atrophy had a mean of JAK3 gene expression fold change 1.77 ± 0.87 with a statistically significant increase of JAK3 gene expression fold change in patients with the presence of epidermal atrophy (p = 0.034). Patients with accentuated hyperpigmentation in histopathology had a mean JAK3 gene expression fold change of 1.77 ± 0.20, with a statistically significant increase in JAK3 gene expression fold change in patients with absent hyperpigmentation than in patients with accentuated hyperpigmentation (p = 0.018, Table 3).

### Relation between JAK gene expression and staging of MF patients using RT-PCR

In early-stage MF, there was a statistically significant increase in JAK1 gene expression fold change than JAK3 gene expression fold change (p < 0.001, Table 4), Supplementary Fig. 3 and 4).

**Table 4:** Relation between JAK fold change and staging in Mycosis Fungoides patients (n = 53).

Staging	N	JAK1 fold change		JAK3 fold change		p
		Mean ± SD	Median (Min. – Max.)	Mean ± SD	Median (Min. – Max.)	
Early stage MF	46	3.10 ± 1.36	2.66 (1.11 – 6.63)	1.55 ± 0.34	1.51 (1.10 – 2.91)	<0.001^a^
Late stage MF	7	3.09 ± 1.37	2.62 (1.30 – 5.21)	3.28 ± 1.43	3.23 (1.25 – 5.23)	0.735
U(P1)		157.50 (0.928)		48.50^a^ (0.002^a^)		

U, Mann Whitney test; P1, p-value for comparing between different categories; p, p-value for Wilcoxon signed ranks test for comparing between JAK1 fold change and JAK3 fold change.

a Statistically significant at p ≤ 0.05.

## Discussion

Mycosis fungoides is the commonest type of primary cutaneous T-Cell lymphoma, accounting for 4% of all non-Hodgkin lymphoma cases. Mycosis fungoides is an indolent disease that could progress from the patch stage to plaque stage, and finally to the tumor stage over a long duration. Risk of lymph nodes and visceral involvement may increase with the lesion's progression.^1^, ^7^

Janus kinase proteins are a family group of intracellular non-receptor tyrosine kinases that includes JAk1, JAK2, JAK3, and Tyk2. JAK1, JAK2, and Tyk2 are expressed ubiquitously in mammals, while JAK3 is primarily expressed in hematopoietic cells. They are involved in the transmission of variable cytokine signals through the JAK–STAT pathway.^4^ Gain of function mutation of JAK proteins could be responsible for a number of myeloproliferative diseases and malignancies.^8^ JAK inhibitors have proven efficacy in the management of some dermatological diseases, including psoriasis and atopic dermatitis.^5^ The present study investigated the role of JAK1 and JAK3 in the pathogenesis of different MF stages, prognosis and severity of MF.

The current study included 53 patients with different clinical subtypes and stages of MF, in addition to 53 healthy controls. MF patients were diagnosed clinically and confirmed by histopathology and cluster differentiation.

In this study, the gene expression of JAK1 and JAK3 in MF using RT-PCR was investigated and correlated with clinical and histopathological parameters of MF. The present study confirmed that the JAK1 and JAK3 gene expression fold change levels using RT-PCR showed a stepwise upregulation with a statistically significant increase of JAK1 and JAK3 gene expression fold change in MF patients compared to healthy controls.

In agreement with this study, Pérez et al. 2015^9^ detected somatic mutations in either JAK1 or JAK3 genes in up to only seven patients and one cell line, from 46 CTCL patients.^9^ Most mutations were within the pseudokinase domain of JAK proteins, which keep the kinase domain inactive until receptor dimerization stimulates transition to an active state.^10^ The treatment with JAK inhibitor activated apoptosis and inhibited DNA synthesis in CTCL cells in their study.^9^

McGirt et al. 2015^11^ found an activating JAK3 point mutation in 1 of the only 5 tested MF tumor samples. They suggested that JAK3 mutation may be a contributing factor to MF. Exposure to low concentrations of tofacitinib induced a significant reduction of cell numbers that was maximized by elevating the JAK3 inhibitor dose, together with a dramatic reduction of phospho-STAT5. Even the cells that showed no JAK3 mutations, demonstrated sensitivity to JAK3 inhibition, indicating that they are still dependent on the JAK3 pathway for their growth and survival.^11^ They were unable to validate their results because of the very low allele frequencies of known activating JAK3 mutations in the other MF samples.^11^

Kiel et al. 2015^12^ demonstrated recurrent gain-of-function mutations targeting JAK1, JAK3, STAT3 and STAT5B in 11% of Sézary syndrome cases. The study revealed the sensitivity of JAK1-mutated primary Sézary syndrome cells to JAK inhibitor treatment.^12^

Regarding JAK expression in other dermatological diseases, JAK proteins were reported to be positively expressed in different common inflammatory skin diseases, including systemic lupus erythematosus, psoriasis, lichen planus, and others.^13^ Using RT-PCR, Awad. et al. 2021^14^ found that JAK1 and JAK3 were overexpressed in acne lesional skin compared to non-lesional skin and controls, owing to the involvement of JAKs in signaling of some cytokines which play a role in the pathogenesis of acne, such as IFN-γ, IL-1, IL-8 and IL-17.^14^

Upregulation of various JAK proteins, including JAK1 and JAK3, is associated with additional malignant cell behavior, tumor growth and metastasis through mediating cell proliferation, regulating the expression of adhesion molecules and the activity of matrix metalloproteinases and macrophages that degrade the extracellular matrix and promote invasion of tumor cell in breast cancer and various digestive tract tumors including liver and gastric cancers.^15^, ^16^

The current study compared JAK1 and JAK3 gene expression fold change levels in different MF clinical types, where the fold changes of JAK1 and JAK3 were more upregulated in tumor stage MF than plaque MF, patch MF, and other clinical subtypes of MF, with statistically significant differences. Hypopigmented MF had the lowest level of JAK3. The tumor stage of classic MF has a high degree of lymphocytic atypia and denser lymphocytic infiltrate than the patch and plaque stages of classic MF, and the higher level of JAK1 and JAK3 fold change in this tumor stage could point to the relation of Janus kinase activation to the severity and progression of MF lesions.

When correlating the gene expression fold change levels of JAK1 and histopathological findings in MF patients, JAK1 fold change was more upregulated in MF patients lacking epidermotropism (2-patients with tumor stage MF) than patients with epidermotropism with no statistically significant difference, and statistically significantly increased in MF patients with heavier and deeper dermal lymphocytes and with the presence of Pautrier microabcesses than patients with papillary dermal infiltrate only and absent Pautrier microabcesses, respectively. JAK3 gene expression fold change was greater in MF patients with absent epidermoptropism and heavier dermal lymphocytes infiltrate than in patients with epidermotropism and papillary dermal infiltrate only.

Ghoreschi et al. 2009^8^ said that multiple signaling events that are mediated by the JAK family are involved in cell growth, development, and differentiation of variable immune and hematopoietic cells, and activating mutation of JAK proteins could be responsible for a number of lymphoproliferative diseases and malignancies.^8^ Additionally, the development of T-lymphocytes from their progenitor cells is strictly dependent on IL-7, and JAK3 is required for signaling of IL-7 receptors, so deficiency of JAK3 can result in defects of lymphocyte development and proliferation.^8^, ^17^ CD4 + T-lymphocytes, which are deficient in JAK3, showed proliferation failure and more susceptibility to apoptosis.^18^ JAK3 is crucial for the differentiation of T helper CD4 + T lymphocytes, not only their proliferation and survival.^19^ This could explain the role of JAK3 in the recruitment and accumulation of T-lymphocytes in MF patients.

The current work found higher JAK1 and JAK3 gene expression fold change in patients with severe atypia than patients with mild and moderate atypia, but with no significant difference (p = 0.220 and p = 0.287, respectively). This correlation may highlight the role of JAK1 and JAK3 in tumorigenesis in MF lesions. In agreement with that, Ruihong Zhao et al. 2024^16^ concluded that aberrant activation of the JAK-STAT pathway may enhance the malignant characteristics of cancer cells, and contribute to uncontrolled proliferation, tumor invasion and metastasis, protection against apoptosis, leading to the development and progression of tumors.^16^ Also, dysregulation of JAK signaling exerts a major role in the malignant transformation of lymphocytes or myeloid cells in a group of hematological malignancies.^8^ L Wu et al. 2023^20^ found that upregulation of JAK1, JAK2, JAK3, and STAT3 in the invasive zone of hepatic tumors could promote local immunosuppression and result in tumor progression.^20^ The previous clarification might help to understand why JAKs are upregulated in skin lesions of MF patients with severe lymphocytic atypia.

In the present study, MF patients with epidermal atrophy showed significantly lower JAK1 gene expression fold change and a significantly higher JAK3 gene expression than those with absent epidermal atrophy (P for JAK1 = 0.002, P for JAK3 = 0.034). Samaka et al. 2019^13^ found a significant association between high JAK1 expression and epidermal atrophy in vitiligo skin.^13^ This could be attributed to the different pathogenesis of both diseases.

In this study, patients with prominent vascularity had a higher JAK3 gene expression fold change than patients with absent vascularity. Yung Wang, et al. 2021^21^ reported that JAK3 could have a regulatory role in the proliferation and regeneration of vascular endothelium for effective repair after vascular injury.^21^ Also, vascular endothelial growth factor production in CTCL, the potent angiogenic factor, was evidenced to be promoted by aberrant activation of JAK3 and c-Jun N-terminal Kinases (JNKs).^22^

In the current results, there was a statistically significant increase in JAK1 gene expression fold change than JAK3 gene expression fold change in early-stage MF (p < 0.001), and in late-stage MF, JAK3 fold change was higher than JAK1 gene expression fold change with no statistically significant difference (p = 0.735). These findings could refer to the more important role of JAK1 in early-stage MF development and the more important role of JAK3 in late-stage MF progression. Similarly, as mentioned before, in early lesions of MF, as in patch stage MF, JAK1 fold change showed a significant increase over JAK3 (p = 0.028), while in advanced MF lesions, as in tumor stage MF, JAK3 fold change was higher than JAK1. Accordingly, enhanced activity of JAK proteins could have a function in the severity and etiopathogenesis of mycosis fungoides.

Guglielmo et al. 2024^23^ summarized the role of different cytokines in CTCL development and progression. They showed that early-stage MF has a high level of Th1 cytokines, such as IL-12 and IFN-γ, and higher serum levels of IL-6. IL-12 could stimulate IFNγ production by T-cells, facilitating Th1 differentiation.^23^ In late-stage MF, there is an increased production of IL-4, which is linked with Th2 cell differentiation, CTCL progression, and the shift from an antitumor to a tumorigenic microenvironment.^23^, ^24^ A higher expression of IL-15 in advanced CTCL stages has also been implicated in the recruitment of CD4+ memory T-cells to the skin, T-cell proliferation, and inhibition of apoptosis.^23^ JAK1 is known to transmit the signals carried by IFN-γ and IL-6, while JAK3 transmits the signal of IL-4, and IL-15.^25^ This could emphasize the role of JAK1 and JAK3 in the pathogenesis of early and late stages of MF, respectively.

The current study concluded that JAKs can be considered valuable not only in diagnostic confirmation but also as markers of MF progression, severity, prognosis, and staging. The study recommends that selective inhibition of JAK1 could be beneficial in the treatment of early-stage MF and JAK3 inhibition might be more effective in late-stage MF treatment.

## ORCID ID

Basma Mourad Mohamed Ali: 0000-0001-5820-5496

Mohamed Labib Salem: 0000-0002-9580-4399

Lamia Elgarhy: 0000-0001-5277-4751

## Data availability statement

Data supporting the findings of this study are available from the corresponding author upon reasonable request.

## Financial support

None declares.

## Authors' contributions

Heba Saed El-Amawy: Study conception; clinical diagnosis; data collection; manuscript writing.

Basma Mourad Mohamed Ali: Clinical supervision; critical revision of the manuscript.

Mohamed Labib Salem: Methodology and analysis.

Lamia Elgarhy: Supervision, editing, and final approval of the manuscript.

## Research data availability

The entire dataset supporting the results of this study was published in this article.

## Conflicts of interest

None declared.
