# Supplementary material for: Evaluation of immunohistochemical and gene expression of Janus kinase1 and Janus kinase3 in the skin of different clinical types of mycosis fungoides patients – Part II: reverse transcriptase–polymerase chain reaction
Source: An Bras Dermatol. 2026 Mar 19;101(2):501300. doi: 10.1016/j.abd.2026.501300 (PMC13015234; doi:10.1016/j.abd.2026.501300)
Supplement: Supplementary file 1 [file mmc1.docx]

**ABD-D-25-00691**

**Supplementary Tables & Box Plots**

**Table 1** Distribution of Mycosis fungoides patients according to clinical data (n = 53).

| **Age (years)** |  |
| --- | --- |
| Mean ± SD. | 39.5 ± 17.7 |
| Median (Min. – Max.) | 44 (5 – 70) |
| **Sex, n (%)** |  |
| Male | 18 (34.0%) |
| Female | 35 (66.0%) |
| **Duration of lesions (in years)** |  |
| < 5 | 34 (64.2%) |
| 5 – 10 | 16 (30.2%) |
| > 10 | 3 (5.7%) |
| Mean ± SD. | 4.82 ± 5.23 |
| Median (Min. – Max.) | 3 (0.08 – 25) |
| **Clinical type, n (%)** |  |
| Classic MF (Patch stage) | 10 (18.9%) |
| Classic MF (Plaque stage) | 9 (17%) |
| Classic MF (Tumor stage) | 3 (5.7%) |
| Hypopigmented MF | 16 (30.2%) |
| Hyperpigmented MF | 7 (13.2%) |
| Poikilodermatous MF | 5 (9.4%) |
| Erythrodermic MF | 3 (5.7%) |
| **Family History, n (%)** |  |
| Negative | 53 (100%) |
| Positive | 0 (0%) |

**Table 2** Distribution of mycosis fungoides patients according to histopathological data (n = 53).

| **Histopathological data** | **Nº of patients (%)** |
| --- | --- |
| Epidermotropism | 51 (96.2%) |
| Dermal lymphocytes infiltrate | 53 (100%) |
| Papillary dermal lymphocytes infiltrate | 50 (94.3%) |
| Papillary and reticular dermal lymphocytes infiltrate | 3 (5.7%) |
| Degree of lymphocytic atypia |  |
| Mild (1) | 31 (58.5%) |
| Moderate (2) | 17 (32.1%) |
| Severe (3) | 5 (9.4%) |
| Pautrier microabcess | 9 (17%) |
| Epidermal atrophy | 5 (9.4%) |
| Prominent vascularity | 9 (17.0%) |
| Basal hyperpigmentation & dermal melanophages | 12 (22.6%) |

**Table 3** Relation between clinical type with histopathological data and staging in mycosis fungoides patients (n = 53).

|  | **Clinical type** | | | | | | | χ^2^ | ^MC^p |
| --- | --- | --- | --- | --- | --- | --- | --- | --- | --- |
|  | **Classic MF (patch stage) (n=10)** | **Classic MF (plaques) (n=9)** | **Classic MF (tumors) (n=3)** | **Hypopigmented MF (n=16)** | **Hyperpigmented MF (n=7)** | **Poikilodermatous MF (n=5)** | **Erythrodermic MF (n=3)** |  |  |
| **Histopathological data** |  |  |  |  |  |  |  |  |  |
| Epidermotropism | 10 (100%) | 9 (100%) | 1 (33.3%) | 16 (100%) | 7 (100%) | 5 (100%) | 3 (100%) | 12.071^a^ | 0.004^a^ |
| **Dermal lymphocytes infiltrate** |  |  |  |  |  |  |  |  |  |
| Papillary dermal lymphocytes infiltrate | 10 (100%) | 9 (100%) | 0 (0%) | 16 (100%) | 7 (100%) | 5 (100%) | 3 (100.0%) | 17.621^a^ | <0.001^a^ |
| Papillary and reticular dermal lymphocytes infiltrate | 0 (0%) | 0 (0%) | 3 (100.0%) | 0 (0%) | 0 (0%) | 0 (0%) | 0 (0%) |  |  |
| **Degree of lymphocytic atypia** |  |  |  |  |  |  |  |  |  |
| Mild (1) | 9 (90.0%) | 3 (33.3%) | 0 (0.0%) | 11 (68.8%) | 5 (71.4%) | 3 (60.0%) | 0 (0.0%) | 25.228^a^ | 0.001^a^ |
| Moderate (2) | 1 (10.0%) | 5 (55.6%) | 0 (0.0%) | 5 (31.3%) | 2 (28.6%) | 2 (40.0%) | 2 (66.7%) |  |  |
| Severe (3) | 0 (0.0%) | 1 (11.1%) | 3 (100.0%) | 0 (0.0%) | 0 (0.0%) | 0 (0.0%) | 1 (33.3%) |  |  |
| Pautrier microabcess | 0 (0%) | 7 (77.8%) | 1 (33.3%) | 0 (0%) | 0 (0%) | 0(0%) | 1 (33.3%) | 24.536^a^ | <0.001^a^ |
| Epidermal atrophy | 0 (0%) | 0 (0%) | 0 (0%) | 0 (0%) | 0 (0%) | 5 (100%) | 0 (0%) | 24.417^a^ | <0.001^a^ |
| Prominent vascularity | 0 (0%) | 1 (11.1%) | 0 (0%) | 0 (0%) | 0 (0%) | 5 (100%) | 3 (100%) | 31.703^a^ | <0.001^a^ |
| Basal hyperpigmentation & dermal melanophages | 0 (0%) | 0 (0%) | 0 (0%) | 0 (0%) | 7 (100%) | 5 (100%) | 0 (0%) | 42.990^a^ | <0.001^a^ |
| **Staging** |  |  |  |  |  |  |  |  |  |
| Early stage MF | 10 (100%) | 8 (88.9%) | 0 (0%) | 16 (100%) | 7 (100%) | 5 (100%) | 0 (0%) | 26.227^a^ | <0.001^a^ |
| Late stage MF | 0 (0%) | 1 (11.1%) | 3 (100%) | 0 (0%) | 0 (0%) | 0 (0%) | 3 (100%) |  |  |

χ^2^, Chi square test; MC, Monte Carlo; p, p-value for comparing between the different categories.

^a^ Statistically significant at p≤0.0.


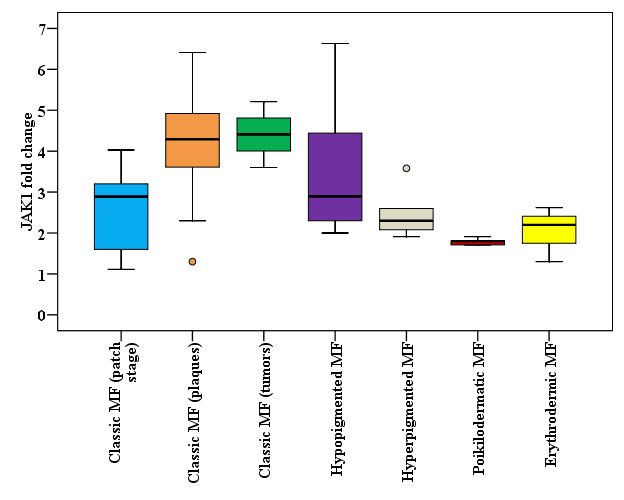


**Figure 1** Relation & Comparison between JAK1 fold change in different MF clinical types (n = 53).

**
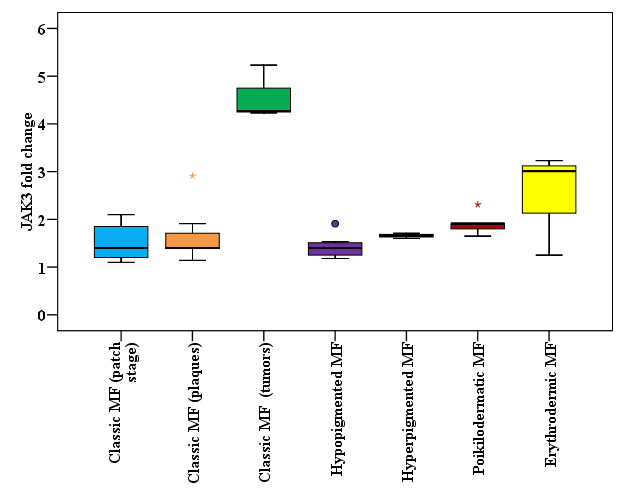
**

**Figure 2** Relation & Comparison between JAK3 fold change in different MF clinical types (n = 53).


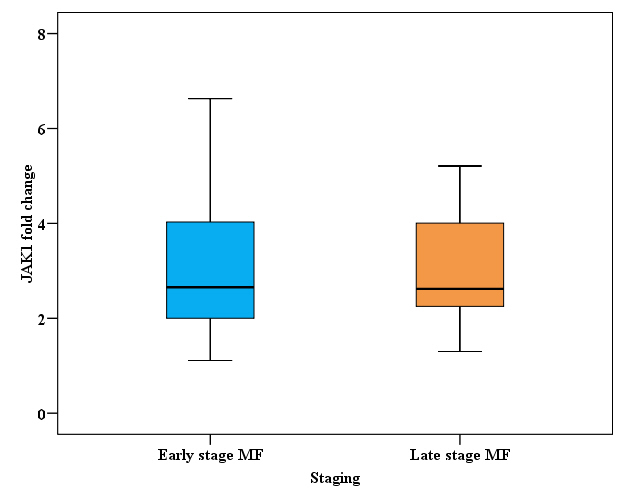


**Figure 3** Relation between JAK1 fold change and staging in MF patients (n = 53).

**
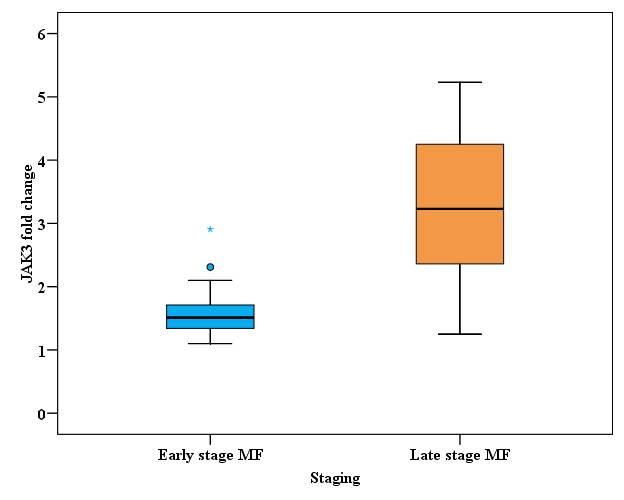
**

**Figure 4** Relation between JAK3 fold change and staging in MF patients (n = 53).
